# Mitochondrial calcium signalling and neurodegenerative diseases

**DOI:** 10.1042/NS20180061

**Published:** 2018-11-16

**Authors:** Elena Britti, Fabien Delaspre, Jordi Tamarit, Joaquim Ros

**Affiliations:** Department of Basic Medical Sciences, University of Lleida, Faculty of Medicine, IRB Lleida, Lleida, Spain

**Keywords:** calcium homoeostasis, mitochondrial permeability transition pore, mitochondria, neurodegeneration

## Abstract

Calcium is utilised by cells in signalling and in regulating ATP production; it also contributes to cell survival and, when concentrations are unbalanced, triggers pathways for cell death. Mitochondria contribute to calcium buffering, meaning that mitochondrial calcium uptake and release is intimately related to cytosolic calcium concentrations. This review focuses on the proteins contributing to mitochondrial calcium homoeostasis, the roles of the mitochondrial permeability transition pore (MPTP) and mitochondrial calcium-activated proteins, and their relevance in neurodegenerative pathologies. It also covers alterations to calcium homoeostasis in Friedreich ataxia (FA).

## Introduction

Calcium is used in many cellular processes and is maintained within the cell as free calcium at low concentrations (approximately 100 nM), compared with extracellular (millimolar) concentrations, to avoid negative effects such as phosphate precipitation [1]. For this reason, cells have adapted buffering strategies by compartmentalising calcium into mitochondria and the endoplasmic reticulum (ER). In mitochondria, the calcium concentration is in the millimolar range, as it is in the ER. Although each cell type displays a series of mechanisms that can affect the intracellular distribution of calcium (to accurately modulate the downstream signalling effects), the interplay between the cytosol, ER and mitochondria is crucial to maintain cellular fitness [2,3]. Mitochondria actively contribute to buffering cellular calcium, but if matrix calcium increases beyond physiological demands, it can promote opening of the mitochondrial permeability transition pore (MPTP) and, consequently, trigger apoptotic or necrotic cell death [4]. The pathophysiological implications of MPTP opening in ischaemia–reperfusion, liver, muscle and lysosomal storage diseases, as well as those affecting central nervous system, for example Parkinson’s disease (PD), Alzheimer’s disease (AD), Huntington’s disease (HD), amyotrophic lateral sclerosis (ALS) have been reported (for a review, see [4] and references therein). Among rare diseases, it has been recently reported that MPTP opening may have a role in Friedreich ataxia (FA) [5]. Mitochondria are dynamic organelles subjected to fusion and fission processes which, when altered, can contribute to developing several neurodegenerative diseases [6]. These diseases include Charcot–Marie–Tooth disease which is related to mutations in mitofusin 2, a mitochondrial outer membrane protein [7], and dominant optical atrophy which is caused by mutations in OPA1 [8], a protein anchored in the mitochondrial inner membrane. Mitofusin 2 and OPA1, along with other proteins, play important roles in fusion/fission processes.

## The homoeostasis Of Ca^2+^ in mitochondria

Processes such as aerobic metabolism, involving the activity of pyruvate-, α-ketoglutarate- and isocitrate-dehydrogenases as well as ATP synthesis, are modulated by the mitochondrial Ca^2+^ concentrations [9,10]. Ca^2+^ uptake by mitochondria was first documented in 1961 by Deluca and Engstrom [11]. Since then, the pathways that maintain the appropriate calcium concentration inside the mitochondria have been revealed to be complex. Calcium exit from the mitochondria was described many years ago by pioneering studies of Carafoli et al. [12,13]. In addition to these fundamental mechanisms, other proteins contribute to maintaining the correct calcium balance in the mitochondria and a brief summary of each one of them is described below.

### Voltage-dependent anion channel

Voltage-dependent anion channels (VDAC1, VDAC2 and VDAC3) are porin-like proteins that are highly abundant in the mitochondrial outer membrane; they have important roles in energy conservation because they allow entry of substrates for the TCA cycle and interact with hexokinase and mitochondrial creatine kinase which generate glucose-6-P and creatine phosphate, respectively [14]. VDACs also play a key role in allowing calcium diffusion into the intermembrane space, which then facilitates uptake into the mitochondrial matrix via the mitochondrial calcium uniporter (MCU), an inner membrane channel (see below) [15]. Most of this calcium comes from the ER in a process that is facilitated by close contact sites, called mitochondria-associated membranes (MAMs), between mitochondria and the ER [16]. In these microdomains, calcium is released from the ER through the inositol-1,4,5-triphosphate receptors (IP3Rs) and ryanodine receptors (RyRs) and enters the ER via the sarcoplasmic/ER calcium ATPase (SERCA), thereby maintaining a fine-tuning of calcium concentration between both compartments [17]. It is important to mention that calcium transfer between IP3R and VDAC depends on the interaction with glucose-regulated protein 75 (GRP75), a molecular chaperone that acts as a bridge between these proteins and facilitates calcium transfer [18]. VDAC proteins interact with antiapoptotic proteins such as Bcl-xL and Bcl-2 to prevent apoptotic cell death [19,20], but upon increase in cell calcium concentration, VDAC expression is increased favouring its oligomerisation; this, in turn, allows the release of cytochrome *c* and apoptosis-inducing factor (AIF) into the cytosol, which promotes the formation of the apoptosome and triggers cell death [21]. The central role of VDAC in neurodegeneration has been described in AD [22,23] and PD [24].

### MCU

Although calcium enters the mitochondria through the VDAC [15,25], the channelling towards the mitochondrial matrix through the inner membrane is mainly accomplished by MCU. Other calcium influx mechanisms include IP3R and RyR1 as already mentioned [17]. MCU is part of the calcium uptake machinery, which also involves MICU1, MICU2, MCU regulator 1 (MCUR1) and essential MCU regulator (EMRE), which, in blue native gels, migrate as a 480-kDa complex [26]. Additional participation of MCUb has also been described [27].

What is the role of these proteins? MCU is the main transporter and can be inhibited by Ruthenium Red, a well-known inhibitor of mitochondrial calcium uptake. Its electrophysiological features were characterised by Clapham et al. [28] and its molecular identity revealed by Rizzuto et al. [29] and Mootha et al. [26]. The mature form is a 35-kDa protein and the pore-forming region consists of eight helices, while the N- and C-termini face the mitochondrial matrix [27]. MICU1, a 54-kDa membrane protein, has two classical EF-hand Ca^2+^-binding domains and interacts physically with MCU [30]. Together with MICU2, it participates in the regulation of MCU activity by binding Ca^2+^ through the EF domains [31]. Other regulators of MCU activity are MCUR1, EMRE and SLC25A23. MCUR1 is an essential component of the MCU complex that acts as a scaffold factor and binds to MCU and EMRE. Cells lacking MCUR1 show severe impairment of mitochondrial calcium uptake and, consequently, of mitochondrial bioenergetics [32]. Regarding the functions of EMRE, recent findings show that it associates with MCU and regulates its activity [33,34]. Such interaction depends on the normal amounts of EMRE which are maintained by mitochondrial proteases. If this proteolytic degradation is inefficient, an excess of EMRE will lead to a constitutive activation of MCU, which promotes mitochondrial calcium overload [34]. Recently, the transient receptor potential 3 (TRPC3), located in the cell membrane, has also been described as being present in mitochondria and as having a role in calcium uptake [35].

### NCLX

Mitochondrial calcium homoeostasis depends not only on the uptake of calcium, but also on the rate at which calcium efflux is actively maintained. This role is accomplished by NCLX, an Na^+^/Ca^2+^ exchanger of the inner mitochondrial membrane [36]. NCLX is related to NCX, the plasma membrane calcium transporter family, but in contrast, it lacks the calcium-binding motifs [37] and, in addition to calcium, NCLX can also transport lithium ions. The relevance of NCLX function was highlighted by Sekler and co-workers [38–40] (after pioneering studies of Carafoli et al. [12]) who showed that NCLX-deficient mitochondria are unable to release calcium into the cytosol. NCLX plays an important role in calcium dynamics in heart [41], brain [42] and pancreatic β-cells [43], and its deletion causes severe myocardial dysfunction and death in mice [44]. It is also involved in regulating the rhythmic contraction of HL-1 cells [45]. Although the mechanisms governing NCLX expression are still unknown, post-transcriptional regulatory events have been described. One of the key proteins related to NCLX function is PINK1 (PTEN-induced putative kinase 1) [46] because, in PINK1-deficient cells, mitochondrial calcium accumulates and increases the formation of reactive oxygen species (ROS). The reason for this is the loss of mitochondrial membrane potential by decreased PINK1 levels, impairing NCLX function. Moreover, phosphorylation by protein kinase A (PKA) at Ser^258^ is a key regulatory element of NCLX function [46]. Other proteins related to NCLX regulation are stomatin-like protein 2 (SLP2), a protein anchored to the mitochondrial inner membrane that, if depleted, contributes to degradation of prohibitins and subunits of the mitochondrial respiratory complexes I and IV [47,48], and μ-calpain, a Ca^2+^-activated protease that also contributes to NCLX degradation upon increased calcium levels in mitochondria [49].

### MPTP

The presence of a regulated, unselective mitochondrial channel was proposed many years ago, although it was generally argued that it was an *in vitro* artifact [50]. The structure and essential components of the MPTP has been a matter of debate for several years, but Bernardi and co-workers at the University of Padova shed some light by showing that it is mainly formed from ATP synthase and regulated by cyclophilin D (CypD), a peptidyl-prolyl *cis-trans* isomerase encoded by the *PPIF* gene [51,52]. According to these findings, CypD interacts with oligomycin sensitivity-conferral protein subunit (OSCP) promoting pore opening. Other proteins such as ANT (adenine nucleotide transporter) and PiC (phosphate carrier) have been also proposed as regulators, if not components, of the MPTP. Recently, it was reported that SPG7 protein (spastic paraplegia 7) may also be a component of such a structure [53], but this finding was questioned [54]. Opening of this pore requires matrix Ca^2+^, which would replace Mg^2+^ at the catalytic site of ATP synthase, triggering a conformational change in catalytic subunits that, in turn, may be transmitted to the lateral stalk subunits via OSCP and facilitating the pore formation [55]. If the pore opening is sustained, it facilitates solute entrance (up to 1.5 kDa ionic and non-ionic compounds) promoting mitochondrial swelling and decreased mitochondrial membrane potential, causing cell death [56,57]. Transient openings have also been reported to play an important function in maintaining calcium concentration (in additon to the role of NCLX; see above) and ROS levels [58]. Post-translational modifications of CypD have also been reported. As an example, in a model of cardiac hypertrophy, CypD becomes acetylated due to decreased activity of the mitochondrial histone deacetylase Sirt3 [59]. Based on these findings, the authors suggest that, when acetylated at Lys^166^, CypD may interact with ANT, increasing the affinity for calcium and, as a consequence, promoting MPTP opening. Since MPTP opening is clearly involved in loss of mitochondrial functions, many MPTP opening inhibitors have been assayed with a view to restoring mitochondrial fitness. Among them, the immunosuppressant drug cyclosporine A (CsA), has proved to be highly efficient in preventing pore opening by interfering with the interaction of CypD with OSCP. Interestingly, benzodiazepine 423 opens the MPTP by interacting with the same site on OSCP [51,60]. More recent compounds such as TRO19622 (olexosime) or TRO40303 [61] have shown neuro- and cardio-protective effects by acting as pore modulators and have been proposed to treat ALS [62]. Furthermore, the drug alisporivir (Debio025), designed to treat hepatitis C, has been repurposed because of its action as a cyclophilin inhibitor, and may be useful to treat collagen VI and Duchenne muscular dystrophies [63,64]. For an extensive review of activators and inhibitors, see [65].

Calcium release from mitochondria to the cytosol through MPTP opening can contribute to activation of calcium-dependent enzymes such as calcineurin, a Ca^2+^-calmodulin-dependent protein phosphatase. Calcineurin activation can affect NFAT (nuclear factor of activated T cells), a transcription factor family of four members (NFATc1–c4) [66]. Although they were formerly believed to be associated with just the immune system, NFAT proteins are expressed in all cell types and they are involved in cardiovascular [67] and nervous system functions [68]. NFAT proteins contain two transcriptional activator domains (at the N- and C-termini): a DNA-binding domain and a regulatory domain (called NHR) which is highly phosphorylated. Calcineurin binds to this NHR domain to activate NFAT, allowing its translocation to the nucleus because the nuclear localisation sequence (NLS) becomes exposed after dephosphorylation [69]. In addition to their role in normal cell physiology, a link to neurodegeneration has also been reported in DRG neurons [70], in AD (for NFATc4) [71,72] and in PD (for NFATc3) [73]. The reason why different NFAT isoforms are involved in these pathologies is unknown but it could be due to their specific regulation properties [74].

### Mitochondrial proteases

Mitochondrial proteases are involved in critical functions such as mitochondrial biogenesis, mitophagy and apoptosis [75]. Some, such as calpains and the ATPases associated with diverse cellular activities (AAA-proteases), are closely related to calcium homoeostasis. Calpains are a family of calcium-dependent cysteine proteases (15 members) [76,77] localised in the cytosol as well as in mitochondria. As general structural features, they have calcium-binding sites and EF-hand motifs that contribute to their activation [78,79]. The concentrations for their activation range from the micromolar (for calpain 1 or μ-calpain) to the millimolar (for calpain 2 or m-calpain) [77]. Calpains 1, 2 and 10 have been described as localising to the mitochondria where they play a role in the degradation of components of the electron transport chain (calpain 10), VDAC (calpain 2), and AIF and NCLX (calpain 1) [49,80–83]. Calpain activation has been described in models of neurodegenerative diseases such as ALS [84,85]. These proteases have an endogenous inhibitor, calpastatin, which regulates calpain activity. It is worth noting that decreased calpastatin levels have been observed in ALS, PD, HD and tauopathy frontotemporal dementia [86], contributing to an abnormal activation of calpains and neuronal death. Consistently, calpastatin overexpression contributed to neuroprotection and increased survival in a mouse model of ALS [87].

The m-AAA proteases have attracted attention because they are involved in specific mitochondrial functions and play important roles in neurodegenerative diseases as they can deregulate calcium homoeostasis [88]. These proteases are ATP-dependent and are usually structured as hexamers; they are inserted in the inner membrane of mitochondria exposing the catalytic domain to the matrix (m-AAA proteases) or to the intermembrane space (i-AAA proteases). m-AAA family members include AFG3L1 and AFG3L2, which are organised as homo-oligomeric or hetero-oligomeric complexes with SPG7, while i-AAA type is formed by subunits of YME1L, a zinc-dependent metalloprotease. AFG3L2 deficiency causes dominant spinocerebellar ataxia type 28 (SCA28) [89], while SPG7 deficiency causes hereditary spastic paraplegia [90]. YME1L deficiency causes optic atrophy 11 [91]. A recent publication has related the role of AFG3L2 to calcium homoeostasis because decreased levels of this protease allow MCU to be constantly activated, causing mitochondrial calcium overload and, as a consequence, MPTP opening and neuronal death [92].

## Mitochondrial calcium and neurodegeneration

From the research findings described in the preceding sections, it is clear that alterations in calcium homoeostasis and signalling are deleterious when it comes to cell function and survival. As mentioned in the introduction, an important role of calcium deregulation in the onset and progression of pathology has been described in AD [93], PD [94], HD [95] and ALS [96]. Although each pathology has specific molecular and cellular features, mitochondrial dysfunction is a common trait in these neurodegenerative diseases, with MPTP opening and altered calcium handling both have roles [97–99] that precede cell death [100]. In AD, the Aβ peptide can be transported into mitochondria via the TOM (translocase of the outer membrane) machinery, can localise to the mitochondrial matrix, interact with specific intra-mitochondrial proteins [98,101] and reduce the activity of respiratory chain complexes III and IV [102]. In addition, the Aβ peptide can promote calcium release from the ER, induce mitochondrial calcium overload [103,104] and consequently, by interaction with CypD, trigger MPTP opening and cell death [105]. Similarly, α-synuclein, the key protein associated with PD, leads to mitochondrial dysfunction and cell death. The role of mitochondria in the pathology of PD is prominent in mice treated with 1-methyl-4-phenyl-1,2,3,6-tetrahydropyridine (MPTP^+^) [106], which is converted into a potent neurotoxin (MPP^+^) that accumulates in dopaminergic neurons and concentrates within mitochondria causing inhibition of complex I [107]. It has been reported that α-synuclein also localises in the mitochondria [108,109] and inhibits complex I activity [110] leading to dose-dependent loss of mitochondrial membrane potential with an associated decrease in phosphorylation capacity [111]. As for AD, there are findings indicating that α-synuclein is involved in calcium release from the ER to the mitochondria due to an increase in the number of MAMs and, consequently, inducing MPTP opening [112]. The role of MPTP in PD is further suggested by the presence of swollen mitochondria in a PD mouse model and the extension of lifespan after genetic ablation of CypD [112].

A role of calcium and mitochondrial dysfunction has been also demonstrated in HD. In rodent models, the disease can be induced with 3-nitropropionic acid (an inhibitor of complex II), which causes behavioural and pathological modifications matching those of HD [113]. Mutant, but not wild-type, huntingtin can interact with the outer mitochondrial membrane and significantly increase the susceptibility of mitochondria to calcium-induced MPTP opening. Indeed, it has been demonstrated that mutant huntingtin induces mitochondrial swelling, MPTP opening, release of cytochrome *c* and subsequent activation of the apoptotic cascade. All these events were completely inhibited by CsA, indicating a role for calcium and MPTP opening in the pathogenesis of this disease [114].

In ALS, the mutant SOD1 protein impairs mitochondrial functions. When mutated, this key protein can aggregate in the mitochondria, bind to the Bcl-2 protein, probably contributing to reducing its anti-apoptotic properties [115], and decrease the activity of complexes I, II and IV of the respiratory chain [116]. Furthermore, motoneurons expressing mutant SOD1 showed an early increase in mitochondrial calcium with loss of mitochondrial membrane potential, mitochondrial swelling and ER overload, followed by an increase in cytosolic calcium [117].

## Calcium dyshomoeostasis in FA

Calcium dyshomoeostasis has also been observed in FA, a neuro-cardiodegenerative pathology [118]. The first evidence of a correlation between calcium and FA was described in the heart, the most affected organ, together with dorsal root ganglia neurons, by Barbeau et al. in 1976 [119]. In this study, intracellular deposits of calcium salts with granular aspect were found in FA myocardial cells together with iron. These results were partially confirmed by Lamarche et al. [120] who observed iron but not calcium deposits in cardiac fibres in FA patients. In another study on the presence of metals in heart tissue of 23 patients, no significant differences were found in total zinc and iron (despite some focal accumulation), while copper and Ca^2+^ levels were respectively lower and higher in the right ventricular wall of FA patients [121]. Although there are some discrepancies, an involvement of calcium in the cardiac pathology of this disease is clearly suggested by the alteration in the T wave of the electrocardiogram, indicating disturbances of calcium–potassium flux. Based on these findings, FA patients were treated with drugs capable of decreasing calcium flux into the cells, such as the β-blocker propranolol and the calcium antagonist verapamil. While a beneficial effect on motor performance and speech has been described for propranolol [122], no significant improvement has been demonstrated for verapamil, possibly because of the advanced stage of the disease [123]. Almost two decades later, it was suggested that a high dose of β-blockers is beneficial for the heart [124].

**Figure 1:**
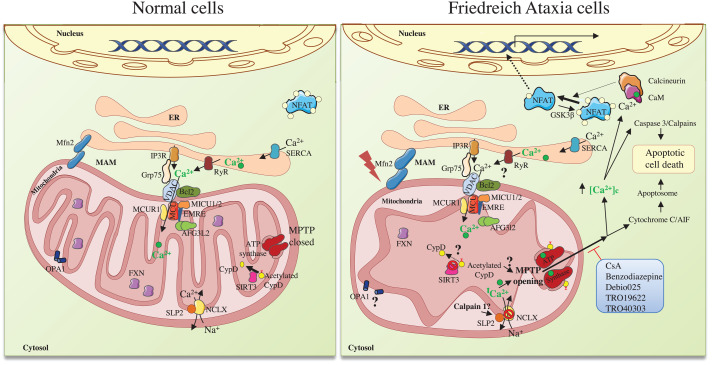
Mitochondrial alterations observed in neuronal models of Friedreich ataxia. The main aspects of mitochondrial dysfunctions and the connections with cytosolic events are summarized.

Apart from its involvement in cardiac pathology, calcium may also have a role in pathogenesis in other cell types. One noteworthy example comes from the Cortopassi and co-workers [125] who rescued oxidant-induced cell death in fibroblasts from FA patients with calcium chelators and inhibitors of apoptosis, suggesting that in FA fibroblasts, calcium dyshomoeostasis could be an early event in the pathology. In this context, Diaz-Nido and co-workers [126] reported that, in frataxin-deficient human neuronal like-cells, cell death was caused by the intrinsic apoptosis pathways with activation of caspase 3 and calpains. As expected, cells were rescued with pan-caspase inhibitors and calpeptin, a calpain inhibitor [126]. In agreement with these results, our laboratory described a frataxin-deficient neuronal model obtained from rat dorsal root ganglia, in which we reported an increase in free intracellular calcium concentration, activation of caspase 3 and calpains, alteration in calcium-mediated signalling pathways and a decrease in NCLX protein levels, together with neurite degeneration and apoptotic cell death (summarized in Figure 1). Interestingly, we also observed decreased NCLX protein levels in frataxin-deficient cardiomyocytes and lymphoblastoid cell lines from patients. NCLX levels were rescued up to the level of controls using either frataxin replacement or treatment with BAPTA, a calcium chelator [5,127]. In addition, frataxin-deficient DRG neurons treated with TAT-BH4 (the anti-apoptotic domain of Bcl-x_L_ fused to the cell-penetrant peptide TAT), BAPTA or CsA, rescued frataxin-deficient DRG neurons from apoptotic cell death, further suggesting a calcium dyshomoeostasis in FA [5,118]. Calcium- and caspase 3-dependent apoptotic cell death was also observed in frataxin-deficient mouse neurons from the embryonic cerebral cortex as well as in a frataxin-depleted mouse cerebellar cortex [128]. In addition, Gonzalez-Cabo and co-workers [129,130] showed that axonal dystrophy in the SH-SY5Y neuroblastoma cell line and in the YG8R mouse model of the disease could be reverted by modulating calcium concentrations. The relationship between mitochondrial and ER calcium balance has been investigated by Navarro and co-workers [131]. By using *Drosophila melanogaster* as an FA model, this work uncovered that the connection between frataxin deficiency and ER stress is mediated by mitofusin-2 and its down-regulation protects from downstream effects of frataxin deficiency. Furthermore, frataxin overexpression in adipocytes causes increased mitochondrial calcium uptake by MCU, up-regulation of the tricarboxylic acid cycle and oxidative phosphorylation, as well as elevated mitochondrial membrane potential and ATP production, without increasing the number of mitochondria [132]. In other words, frataxin increased the mitochondrial calcium buffer capacity. To sum up, frataxin deficiency in several models of the disease, including human neuronal-like cells, fibroblasts, DRG neurons, cerebral cortex neurons and cerebellum, led to calcium deregulation and caspase 3 activation, a common mechanism of apoptotic cell death.

Although the initial steps of the process through which decreased levels of frataxin leads to neurodegeneration are still unknown, the above-reported findings clearly point to an important role of calcium deregulation in the pathophysiology of FA. Therapeutic strategies that impact on the proteins responsible for calcium overload and its consequences could be of great value for developing a cure for the disease.

## Conclusion

Mitochondrial calcium homoeostasis clearly contributes to cellular fitness and survival. Maintenance of accurate calcium levels is highly regulated by mitochondrial proteins that are connected, either directly or via other proteins, to ER calcium stores. As described in this review, prior to cell death, mitochondrial calcium deregulation could be the consequence of increased influx (MCU complex), or decreased efflux (Na^+^/Ca^2+^ exchanger) or altered capacity for calcium buffering caused by mitochondrial damage, such as MPTP opening. Calcium deregulation would then lead to several neurodegenerative processes that resulted in cell death. Thus, identifying specific targets to maintain calcium balance and mitochondrial function appears to be of paramount importance in preventing, alleviating or even curing neurodegenerative diseases.

## Abbreviations

AD: Alzheimer’s disease
AIF: apoptosis-inducing factor
ALS: amyotrophic lateral sclerosis
ANT: adenine nucleotide transporter
CsA: cyclosporine A
CypD: cyclophilin D
EMRE: essential MCU regulator
ER: endoplasmic reticulum
FA: Friedreich ataxia
HD: Huntington’s disease
IP3R: inositol-1,4,5-triphosphate receptor
MAM: mitochondria-associated membrane
MCU: mitochondrial calcium uniporter
MCUR1: MCU regulator 1
MPTP: mitochondrial permeability transition pore
NCLX: mitochondrial sodium-calcium exchanger
NFAT: nuclear factor of activated T cell
OSCP: oligomycin sensitivity-conferral protein subunit
PD: Parkinson’s disease
PINK1: PTEN-induced putative kinase 1
ROS: reactive oxygen species
RyR: ryanodine receptor
SPG7: spastic paraplegia 7
VDAC: voltage-dependent anion channel
